# Supplementary figures and images for: The Natural Compound CalebinA Suppresses Gemcitabine Resistance and Tumor Progression by Inhibiting Angiogenesis and Invasion Through NF-κB Signaling in Pancreatic Cancer
Source: Nutrients. 2025 Aug 14;17(16):2641. doi: 10.3390/nu17162641 (PMC12389118; doi:10.3390/nu17162641)

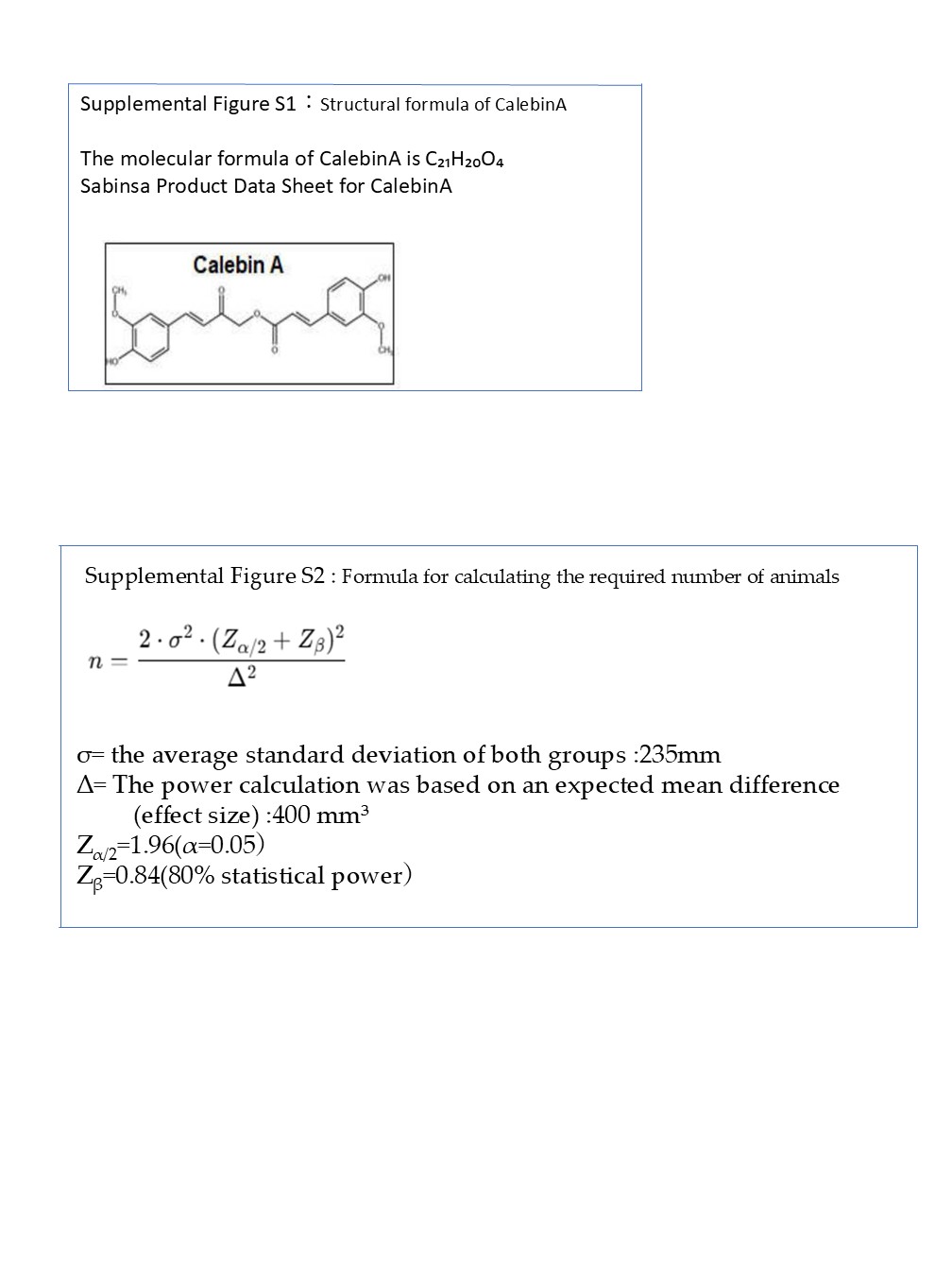

Supplement: Supplementary file 1 [file nutrients-17-02641-s001.zip › nutrients-3782441-supplementary.jpg]
